# Exploring Bridge Symptoms in Postpartum Women With Comorbid Postpartum Depression and Postpartum Post-Traumatic Stress Disorder

**DOI:** 10.1155/da/5629630

**Published:** 2025-08-21

**Authors:** Wei Wei, Meidi Xiong, Miao Tian, Ping Liu, Chunhua Zhou, Huijun Cheng, Chunhua Zhang

**Affiliations:** ^1^Department of Obstetrics, Zhongnan Hospital of Wuhan University, 169, East-Lake Road, Wuhan 430071, China; ^2^Nursing Department, Zhongnan Hospital of Wuhan University, 169, East-Lake Road, Wuhan 430071, China; ^3^Department of Pediatrics, Zhongnan Hospital of Wuhan University, 169, East-Lake Road, Wuhan 430071, China; ^4^Department of Neurosurgery, Zhongnan Hospital of Wuhan University, 169, East-Lake Road, Wuhan 430071, China

**Keywords:** bridge symptoms, postpartum depression, postpartum post-traumatic stress disorder, postpartum women, symptom network

## Abstract

**Background:** Postpartum depression (PPD) and postpartum post-traumatic stress disorder (PP-PTSD) are prevalent among women. However, the specific symptoms that serve as bridges remain unknown between these two disorders.

**Aim:** The objective of this study is to establish a symptom network model for PPD and PP-PTSD and investigate the bridge symptoms and their interrelationships in cases of comorbid PPD and PP-PTSD.

**Methods:** A cross-sectional study was conducted at a tertiary hospital in Wuhan from March 2024 to November 2024. PPD was evaluated using the Edinburgh Postnatal Depression Scale, and PP-PTSD was measured using the Chinese version of the Perinatal PTSD Questionnaire. The “Postpartum Depression–Postpartum Post-traumatic Stress Disorder” network model was constructed and analyzed using R software version 4.2.3.

**Conclusion:** Healthcare professionals should focus on the severe bridge symptoms reported by postpartum women. To enhance awareness and alleviate anxiety levels, it is advisable to implement positive psychological interventions.

**Summary**

• Issue
  ◦ Postpartum depression (PPD) and postpartum post-traumatic stress disorder (PP-PTSD) have both been identified as unique risk factors for adverse psychiatric outcomes in women during and after pregnancy, exhibiting a higher prevalence and causing severe detriment.
• What is already known
  ◦ A multitude of factors can influence the comorbidity and prevalence of PPD and PP-PTSD. The relationship between these structures was briefly elaborated by performing independent analyses of PPD, PP-PTSD and other covariates.
• What this paper adds
  ◦ This study constructs a symptom network delineating PPD and PP-PTSD in postpartum women, exploring the bridging relationship and symptoms between PPD and PP-PTSD. This approach offers novel insights into the development of targeted interventions.

## 1. Introduction

Postpartum depression (PPD) can manifest at any point within the first year postpartum [1], during which they are highly susceptible to multiple physiological and psychological stressors. Studies have identified PPD and postpartum post-traumatic stress disorder (PP-PTSD) as distinct risk factors contributing to maternal psychopathology [2]. The incidence of PPD is estimated to be approximately twice that of depression at other life stages, yet it frequently remains undetected and untreated. PPD can manifest at any point within the first year postpartum and may persist for several years [3]. The global prevalence of PPD is approximately 17.22%, varying across different geographic regions, ranging from 11.11% in Oceania to as high as 39.96% in Southern Africa [3]. Notably, in China, the reported prevalence of PPD stands at approximately 16%, exhibiting substantial regional disparities, with rates spanning from 9% to 31% across various provinces and cities [4]. However, early identification and diagnosis of PPD remain a major challenge, with over 50% of affected women remaining undiagnosed [5]. PPD has profound negative impacts on postpartum recovery, characterized by emotional instability, memory decline, loss of appetite, emotional numbness, anxiety, irritability, and frequent crying episodes. Additionally, affected mothers often experience overwhelming guilt and helplessness. In extreme cases, they may develop thoughts of self-harm or even harming their infant, with the most severe cases potentially culminating in suicidal tendencies [3, 6]. Alarmingly, research has confirmed that suicide is a leading cause of direct maternal mortality within the first year postpartum. The maternal suicide rate in Japan is reported to be 8.7 per 100,000 live births, while in Iran, it is estimated at 2.64 per 100,000 [7, 8]. This tragic phenomenon inflicts profound and lasting psychological and emotional trauma not only on the affected women but also on their infants, partners, and families. Moreover, it places a considerable burden on healthcare systems and incurs substantial economic costs to society [9, 10].

Similarly, PP-PTSD is another prevalent mental disorder during the postpartum period. The prevalence of PP-PTSD varies greatly across different countries, with reported rates of 5.1% in Western countries [11, 12], and 6.1% in China [13]. The core symptoms of PP-PTSD include heightened stress responses (persistent hyperarousal), avoidance of trauma-related stimuli (such as avoiding childbirth-related situations or interactions with the infant), and intrusive experiences (reexperiencing negative emotions associated with the birth-related trauma) [14]. Studies have demonstrated that PP-PTSD can lead to a range of adverse outcomes, including disrupted mother–infant interactions, which may impair maternal-infant attachment, as well as negative consequences for the partner and the overall family dynamic [15]. Despite increasing global attention on PPD and PP-PTSD—particularly their long-term impact on maternal and infant health—research in China, though advancing, remains relatively limited in systematically exploring the relationship between these two conditions. Notably, studies have reported a high comorbidity rate between PPD and PP-PTSD, with 90.4% of women diagnosed with PP-PTSD also experiencing PPD, and 31.5% of those with PPD having a history of PP-PTSD [16]. This high comorbidity rate underscores the profound interconnection between the two disorders, suggesting that PP-PTSD may predispose individuals to the development of PPD, while the presence of PP-PTSD could, in turn, exacerbate or prolong the course of PPD.

A wide range of risk factors for PPD have been identified in the literature. Among these, unintended pregnancy is a major contributor, as women who conceive unexpectedly are often unprepared for motherhood. The disruption of their work and daily routines may increase their susceptibility to depression [12, 17, 18]. Furthermore, women who undergo cesarean section (C-section) may experience a higher psychological burden due to postoperative pain, uncertainty regarding wound healing, and functional limitations during recovery. Additionally, the inability to experience vaginal delivery may lead to self-doubt regarding reproductive capability and even feelings of guilt for not giving birth “naturally”, which could hinder mother-infant bonding and elevate the risk of PPD [8, 19]. Studies have also shown that maternal PPD rates are notably lower in families where husbands are actively involved in infant care, likely due to the emotional support and enhanced family dynamics provided by paternal participation [3, 20]. Moreover, research suggests that mothers who adopt a mixed feeding approach (both breastfeeding and formula feeding) [8, 21] and as well as those in the postpartum period of 3–6 months are at a higher risk of developing PPD [22]. Similarly, various risk factors for PP-PTSD have been identified. Compared to employed women, unemployed mothers may face greater psychological stressors due to financial stress and reduced social support. The interplay of economic uncertainty and the physical and emotional demands of childcare may subtly accumulate as underlying stressors, potentially compromising psychological resilience and increasing the risk of PP-PTSD [23]. Likewise, low-income families, due to concerns over childcare costs and household expenses, are more vulnerable to PP-PTSD [23]. However, in China, no studies have yet explored these two clinical conditions in conjunction.

PTSD and PPD are highly correlated and often co-occur among postpartum women, with substantial overlap in both clinical presentation and underlying mechanisms. Studies show that 65% of women diagnosed with postpartum PTSD also exhibit depressive symptoms, while approximately 22% of those with PPD meet criteria for PTSD [24]. This comorbidity may stem from acute medical events—such as emergency cesarean sections, severe postpartum hemorrhage, or the intense sense of helplessness and loss of control during childbirth—which often trigger trauma responses. These responses, characterized by hypervigilance, intrusive memories, and emotional withdrawal, substantially disrupt emotional regulation and the capacity for intimate bonding, laying a psychological foundation for the development of depressive symptoms. Conversely, women with preexisting difficulties in emotional regulation or a vulnerability to depression may lack the psychological resources to effectively process and integrate childbirth-related stressors, making them more susceptible to persistent traumatic responses [25]. Research has shown that early postpartum trauma symptoms account for more than a quarter of the variance in depressive symptoms observed at 3 months postpartum [26]. This substantial overlap is not merely psychological but also materializes in the mother-infant relationship. Trauma responses frequently lead to maternal behaviors, including avoidance, emotional detachment, or hyper-vigilance towards the infant. All these behaviors impede the establishment of a secure attachment. Similar disruptions are also commonly observed in women with PPD, further underscoring the shared functional impairments associated with both disorders.

Most previous studies have primarily assessed the comorbidity, prevalence, and risk factors of PPD and PP-PTSD. While independent analyses of PPD, PP-PTSD, and other covariates provide insights into how these symptoms interact, they offer only a fragmentary understanding of the intricate relationships between these constructs [13, 23]. Symptom network theory, which conceptualizes psychiatric disorders as interconnected symptom networks rather than distinct, isolated conditions, offers a novel approach to understanding these relationships. By constructing multidimensional symptom networks and identifying bridge symptoms that connect the two clusters, researchers can gain deeper insights into their interconnectivity, ultimately paving the way for more precise and individualized interventions [27]. Previous studies have developed symptom networks across various populations, including cancer patients, individuals living with HIV, and those with chronic illnesses, to capture the complex interplay among different symptoms [27–29].

To enhance the precision and personalization of interventions, thereby improving the effectiveness and efficiency of treatment strategies, identifying bridge symptoms is crucial. However, limited research has focused on multidimensional symptom networks to pinpoint these bridge symptoms and assess the interconnections between PPD and PP-PTSD among postpartum women. Currently, there still exists a substantial disparity in our understanding of the specific bridge symptoms linking PPD and PP-PTSD in this population. Empirical evidence is needed to develop personalized and targeted psychological interventions for postpartum women experiencing these conditions. Therefore, the aims of this study are to: 1) Construct a symptom network of PPD and PP-PTSD in postpartum women. 2) Identify bridge symptoms and explore the interconnections between PPD and PP-PTSD.

## 2. Methods

### 2.1. Study Design and Settings

This cross-sectional observational study was designed to apply symptom network analysis to identify and scrutinize bridge symptoms that connect PPD and PP-PTSD among postpartum women. Data will be executed via the administration of questionnaires, and analyzed using the network model.

### 2.2. Participants

Participants were recruited from a tertiary hospital in Wuhan, China, between March and November 2024. Inclusion criteria stipulated that participants must be married women aged 20 years or older, with an uncomplicated postpartum status, a surviving newborn, long-term residency in China, and adequate cognitive capacity to independently complete the questionnaire. Exclusion criteria encompassed the presence of severe complications or comorbidities, unmarried status, widowhood, divorce, termination of pregnancy at or beyond 28 weeks of gestation, or the existence of severe mental or physical pathologies. Researchers screened eligible participants, explained the study's purpose and procedures to both the mothers and their families, obtained informed consent, and assisted them in signing the consent form. This study was approved by the Institutional Review Board of Zhongnan Hospital of Wuhan University (Approval No. [2024280K]).

### 2.3. Data Collection

Upon identifying eligible participants, the researchers provided a comprehensive elucidation of the study's purpose, significance, and questionnaire instructions before data collection, allowing sufficient time for participants to consider their involvement. The questionnaire was meticulously crafted using the online platform “Wenjuanxing” and was distributed via a QR code, accessible either via WeChat or on-site scanning. To ensure data quality, all questions were set as mandatory, and the estimated completion time was 10–15 min. Upon the submission of each questionnaire, two researchers independently reviewed each questionnaire to assess its completeness and accuracy, removing responses that were incomplete, contained errors, or displayed obvious response patterns. A total of 610 questionnaires were distributed, and 580 were returned. After the initial exclusion of 30 blank or duplicate responses, two researchers independently reviewed the remaining questionnaires. Questionnaires were deemed invalid and excluded based on the following criteria: (1) incomplete responses; (2) logical inconsistencies or repeated selection of the same option; and (3) abnormal completion times (less than 3 min or more than 30 min). Ultimately, 550 valid questionnaires were retained, yielding an effective response rate of 90.16%.

## 3. Measures

### 3.1. Demographic Characteristics Questionnaire

Demographic data, along with scores for PPD and PP-PTSD, were collected. Among these, postpartum duration, age, education level, employment status, only child status, average monthly household income, pregnancy intent, conception method, history of miscarriage, pregnancy complications, fear of labor, first childbirth, newborn delivery status, postpartum hemorrhage, newborn admitted to nicu, feeding method, baby's gender matches expectation, infant care arrangement, husband's involvement in infant care, and mode of delivery were treated as categorical variables, while the scores of PPD and PP-PTSD were treated as continuous variables.

### 3.2. The Edinburgh Postnatal Depression Scale

The Edinburgh Postnatal Depression Scale, developed by Cox et al. [30] in 1987, consists of 10 items that assess various aspects of mood, including enjoyment, guilt, anxiety, fear, insomnia, coping ability, sadness, crying, and self-harm. The scale uses a 4-point Likert scale, with responses ranging from “never” to “always,” scored from 0 to 3. The total score ranges from 0 to 30, with a higher score indicating more severe depression. A score of 13 is considered the threshold for postpartum depressive symptoms, with a score of ≥13 indicating the presence of PPD. In this study, the Cronbach's α coefficient for the EPDS among postpartum women was 0.847, indicating good internal consistency.

### 3.3. Perinatal Post-Traumatic Stress Disorder Questionnaire Scale

The Perinatal Post-traumatic Stress Disorder Questionnaire Chinese Edition was used to assess symptoms of PP-PTSD [31]. The Perinatal Post-traumatic Stress Disorder Questionnaire Chinese Edition is a self-report scale consisting of 14 items, which assess three key dimensions of PP-PTSD: intrusive recollections, avoidance, and hyperarousal. It is based on the diagnostic criteria from the Diagnostic and Statistical Manual of Mental Disorders and is widely used for evaluating PTSD in perinatal women. The scale uses a 4-point Likert scale, ranging from “not at all” to “more than once a month” with scores ranging from 0 to 4 for each item. The total score ranges from 0 to 56, with a score of ≥19 indicating the presence of PP-PTSD symptoms. In this study, the Cronbach's *α* coefficient for the Perinatal Post-traumatic Stress Disorder Questionnaire Scale was 0.896, indicating good reliability for measuring PP-PTSD symptoms in the postpartum period.

### 3.4. Data Analysis

Statistical analysis was performed using *R* version 4.2.3 software. Continuous variables with a normal distribution were described as means (*x*^2^ ± s), while skewed continuous variables were described as median (Interquartile Range: Q1, Q3). The symptom network was constructed using the qgraph package in *R*, with the EBICgl asso function and Spearman correlation analysis. The network was simplified by using the least absolute shrinkage and selection operator to reduce weak edges. Symptoms were represented as nodes in the network, and edges between nodes represented partial correlation coefficients. Thicker edges indicated stronger correlations between two symptoms. Centrality measures for the model were calculated as follows: Betweenness Centrality measures the frequency with which a node lies on the shortest path between other nodes. A higher betweenness indicates stronger regulatory capacity for other nodes. Closeness Centrality is the inverse of the sum of distances from a node to all other nodes, indicating that nodes with higher closeness are more tightly connected to the network. Strength Centrality is the sum of the weighted values of all the edges connected to a node, with a higher strength indicating greater influence on the overall network. Finally, the Correlation Stability Coefficient (CS-C) was calculated using the Bootnet package in *R* to assess the stability of centrality indices. A CS-Cs of at least 0.25 is considered acceptable, and a value ≥0.5 indicates sufficient stability. Non-parametric bootstrapping was used to calculate 95% confidence intervals (CIs) to determine the accuracy of edge weights. Smaller 95% CI intervals indicate higher accuracy of the edge weights. We conducted univariate and multivariate analyses using multiple linear regression to evaluate the influence of various variables on PP-PTSD and PPD. The table lists the regression coefficients for each variable, including the results from univariate and multivariate analyses, along with the corresponding *p*-values to determine the statistical significance of the variables.

## 4. Results

### 4.1. Participant Characteristics

Table 1 presents the characteristics of the sample. The majority of participants were aged ≤35 years (465, 85%), were non-only children (337, 61%), had a monthly income exceeding 5000 yuan (436, 79%), had planned pregnancies (434, 79%), conceived naturally (506, 92%), had no history of miscarriage (386, 70%), and had no complications (436, 79%). Among the participants, 251 (46%) had a bachelor's degree, and 132 (24%) were government employes. The mean score for PPD among postpartum women was 14.38 ± 3.98, while the mean score for PP-PTSD was 20.19 ± 10.68.

### 4.2. Univariate Analysis

Table 2 shows the results of the univariate analysis indicated that PPD showed statistically significant associations with postpartum duration (*p*  < 0.05). Specifically, pregnancy intent (*p*  < 0.05), feeding method (*p*  < 0.05), husband's involvement in infant care, and mode of delivery (*p*  < 0.05). No statistically significant associations were identified with other variables (*p*  > 0.05). Regarding PP-PTSD, no statistically significant associations were detected with postpartum duration, age, education level, only child status, pregnancy intent, conception method, history of miscarriage, pregnancy complications, fear of labor, first childbirth, newborn delivery status, postpartum hemorrhage, newborn admitted to NICU, feeding method, baby's gender matches expectation, infant care arrangement, husband's involvement in infant care, or mode of delivery (*p*  > 0.05). Nevertheless, statistically significant associations were found with employment status (*p*  < 0.05) and average monthly household income (*p*  < 0.05).

### 4.3. Multivariate Analysis

In this research, a multiple regression analysis was conducted to investigate the influencing factors of PPD and PP-PTSD, with PPD and PP-PTSD scores of the women serving as the dependent variables. Based on the variables that exhibited statistically notable disparities in the univariate analysis, corresponding independent variables were selected. The findings revealed that the predictors for PPD encompassed a postpartum duration of 3–6 months (5.13 [1.62 to 8.65], *p*=0.004) and cesarean section (0.70 [0.02 to 1.38], *p*=0.044). Conversely, the predictors for PP-PTSD included unemployment status, and household monthly income ≤3000 yuan (6.93 [2.30 to 11.57], *p*=0.003).

### 4.4. PPD—PP - PTSD Network Analysis

**Figure 1:**
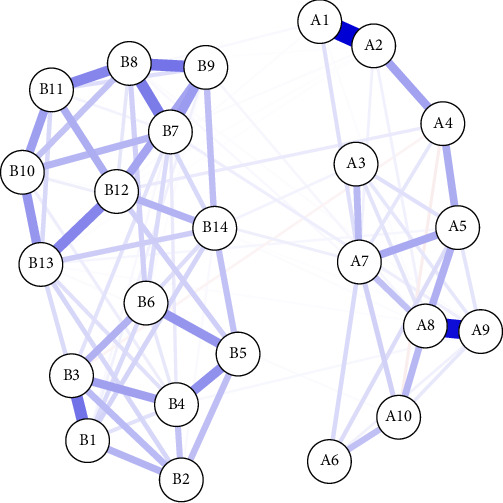
Network relationships in postpartum depression and postpartum post-traumatic stress disorder.

The network analysis of PPD—PP-PTSD includes two key indicators: PPD and PP-PTSD. The network structure is presented in Figure 1, and the node labels are listed in Table 3, revealing relatively stronger connections among nodes within each respective scale. Among the associations between PPD and PP-PTSD, the three strongest connections were observed between “B12 (Harder to concentrate)” and “A4 (Unreasonable anxiety)” (*r* = 0.201), “B9 (Hard to feel love)” and “A8 (Sad and miserable)” (*r* = 0.228), and “B7 (Lost interest)” and “A9 (Hard to feel love)” (*r* = 0.225).

### 4.5. Bridge Strength

**Figure 2:**
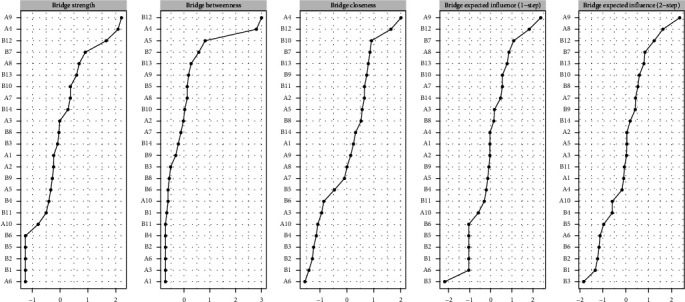
Bridge strength of the attitude towards mental problems-doctor–women relationship network.

Based on bridge strength values, we identified three key bridge symptoms. The highest bridge strength was observed in PPD symptom “A9 (unhappy and cry)” with a value of 0.375, followed by “A4 (Unreasonable anxiety)” from PPD and “B12 (Harder to concentrate)” from PP-PTSD, with bridge strengths of 0.358 and 0.357, respectively (Figure 2). To address the symptoms of bridges, we designed some intervention measures (such as mindfulness training to alleviate anxiety, social support therapy to improve emotional connections).

### 4.6. Network Examination

**Figure 3:**
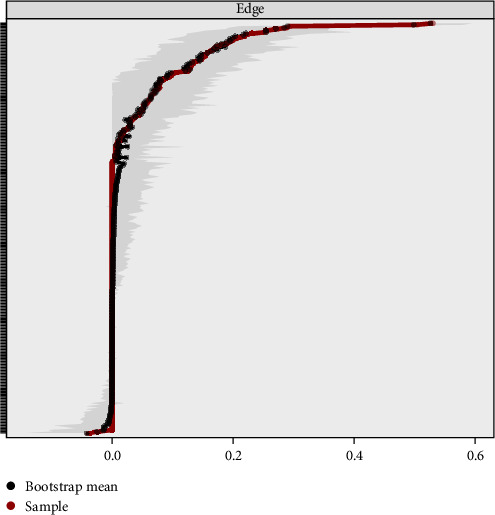
Accuracy of edge weight.

**Figure 4:**
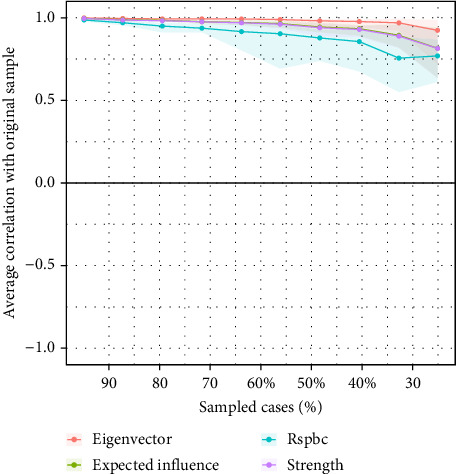
Stability of bridge strength.

**Figure 5:**
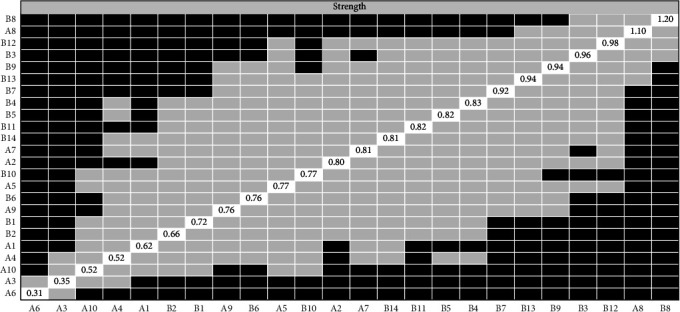
Difference test of bridge strength. The small black box represents the difference in bridge strength between the two corresponding nodes is statistically significant (*p*  < 0.05), and the small gray box represents the difference in the values of the two corresponding variables is not statistically significant (*p*  > 0.05).

**Figure 6:**
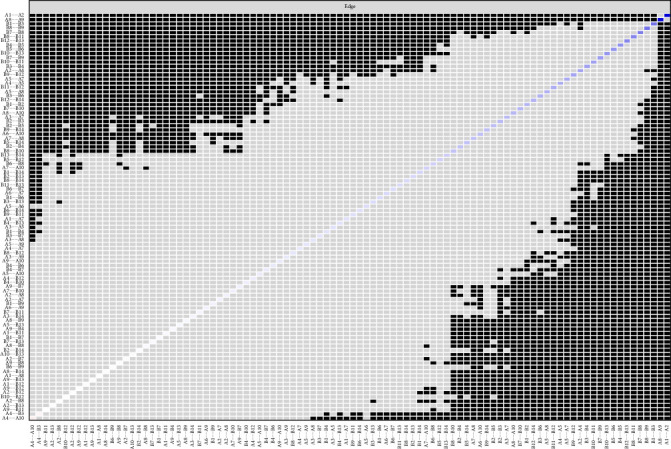
Difference test of edge weight. The small black box represents the difference in edge weight between the two corresponding nodes is statistically significant (*p*  < 0.05), and the small gray box represents the difference in the values of the two corresponding variables is not statistically significant (*p*  > 0.05).

We conducted accuracy analysis, stability analysis, and difference testing for the network. As shown in Figure 3 (left), the accuracy of edge weights was assessed using bootstrapping, with the 95% CIs appearing relatively narrow, indicating a high level of estimation precision.  Figure 4 presents the CS-Cs for various centrality measures: the CS-C for bridge strength is 0.595, for eigenvector centrality is 0.749, and for RSPBC random spanning tree betweenness centrality (RSPBC) is 0.516. According to previous research, a CS-C value should ideally exceed 0.50, with 0.25 as the minimum acceptable threshold. Therefore, all reported centrality measures in this study demonstrate adequate stability.  Figure 5 presents the results for bridge strength, and Figure 6 displays the outcome of the difference tests for edge weights. In both figures, black squares indicate statistically significant differences (*p*  < 0.05), while gray squares denote nonsignificant differences (*p*  > 0.05).

## 5. Discussion

As is well known, the global prevalence of PPD is approximately 17.22%. The prevalence of PP-PTSD is 6.1% in China, and the comorbidity rate between PPD and PP-PTSD has been reported a high rate. This study aims to develop a symptom network to explore bridge symptoms and the interconnections between PPD and PP-PTSD in affected women.

First, research has pinpointed multiple factors in the prominent characteristics of PPD and PP-PTSD. The degree of depression among women from 3 and 6 months postpartum was obviously higher than those within the first month postpartum. This finding is in line with a study carried out at a teaching hospital in Guangzhou, China, which surveyed the depression levels of 122 mothers at multiple postpartum time points. The study revealed that the mothers' depression scores gradually increased from 2 to 3 days postpartum to 6 months, reaching a peak between 3 and 6 months [22]. This may be attributed to the crucial phase of psychological adaptation for mothers. During this period, the integrated impacts of role reshaping, increasing parenting pressures, and dynamic changes in social support pose substantial challenges to emotional regulation and psychological resilience, ultimately resulting in a notable elevation of depression levels. Furthermore, the mode of delivery exerted a substantial influence on postpartum mental well-being. This research identified a notable correlation between cesarean delivery and an elevated risk of PPD. A similar conclusion was confirmed in a cross-sectional study conducted in the Ankulia region of South Orissa, where the prevalence of depression was remarkably higher in the cesarean delivery group compared to vaginal delivery group [19]. This finding further supports the role of cesarean delivery as a potential risk factor in postpartum mental health.

In addition, compared with government employed and self-employed, unemployed women are more likely to suffer from PP-PTSD. This increased risk may be attributed to the sharp increase in postpartum family economic pressure. Unemployed women lack a stable economic source and have fewer social opportunities for contact and activities, leading to diminished self-worth and reduced social support. These factors can contribute to the development of PP-PTSD. The results align with previous studies indicating that unemployed women are more susceptible to develop PTSD [32]. As expected, mothers with a total household income of ≤3000 RMB were also more likely to develop PP-PTSD compared to those with higher incomes. A study on comorbid trajectories of PPD and PTSD in mothers with a history of childhood trauma found that over 30% of affected individuals were in the lowest income bracket (below £25,000), and nearly half were single, which may contribute to their depressive symptoms [2]. When caring for young infants, accumulated economic pressures can profoundly undermine postpartum mental health. This suggests that postpartum mental health interventions should consider a comprehensive range of factors, including an individual's occupational identity, economic status, and history of trauma, to provide more effective support for different groups.

Through symptom network analysis, we identified “Unreasonable anxiety” and “Harder to concentrate” as key bridge symptoms between the clusters of PPD and PP-PTSD. The network also revealed that the correlations between “Unreasonable anxiety” and “Harder to concentrate,” as well as between “Unhappy and cry” and “Lost interest,” and “Sad and miserable” and “Hard to feel love,” were stronger compared to other relationships. Additionally, when analyzing the bridge strength of the “PPD and PP-PTSD” network, we found that “Unhappy and cry,” “Unreasonable anxiety,” and “Harder to concentrate” emerged as bridge nodes between PPD and PP-PTSD. Notably, “Unhappy and cry” exhibited the highest bridge strength, suggesting that these symptoms could serve as bridges between the clusters.

In complex networks, bridge nodes play a crucial role. Although causal relationships between symptom interactions cannot be inferred, these nodes serve as central links that sustain the co-occurrence and interaction of different symptom clusters [27, 33]. This finding is particularly noteworthy because many postpartum women may simultaneously experience symptoms of both PPD and PP-PTSD. Due to the fear of childbirth experiences, intolerance to uncertainty regarding postpartum recovery and infant health outcomes, and the sudden changes in postpartum body image and roles, many postpartum women may simultaneously experience symptoms of both PPD and PP-PTSD [34, 35]. Additionally, previous studies have reported that PPD and PP-PTSD may mutually influence each other to some extent. This interaction could gradually evolve into a more complex mental health condition, ultimately leading to comorbidity between PPD and PP-PTSD symptoms [36]. The newborn's birth often receives great attention, while the psychological fluctuations and emotional downturns experienced by the mother after childbirth are often overlooked, becoming a hidden root cause of mental health issues in postpartum women [37]. Multiple studies have shown that postpartum women may experience various emotions, which are seen as defense mechanisms protecting them from stress and trauma [38]. However, an excessive number of comorbid symptoms can increase the connectivity of the network, and network connectivity is a key characteristic in detecting changes in prognosis [39]. Therefore, interventions targeting bridge symptoms may be more effective and precise than overall psychological therapy interventions in altering the interactions between PPD and PP-PTSD networks [40].

This study also found strong correlations between “Unreasonable anxiety”, “Harder to concentrate”, “Unhappy and cry”, “Lost interest”, “Sad and miserable”, and “Hard to feel love.” These six symptoms form the “bridge portion” of the entire network, suggesting that symptoms in the bridge portion are more involved in clustering PPD and PP-PTSD symptoms, maintaining the integrity of the entire interactive network. Although this study analyzed cross-sectional data and cannot determine the true causal relationships between symptoms, predictive indicators can offer hypotheses for causal interference in complex networks [40, 41]. When postpartum women report highly severe symptoms in the bridge portion, healthcare providers should be vigilant in identifying psychological changes, ensuring that these women receive adequate emotional and psychological care and support.

To alleviate unreasonable anxiety and enhance concentration in postpartum women, various psychological therapies currently used to treat depression and PTSD can be applied. Psychotherapy spans multiple fields, with different therapeutic approaches often targeting specific symptoms to achieve better therapeutic outcomes [42]. Previous studies have shown that music therapy combined with kangaroo care may be more effective in reducing unreasonable anxiety in postpartum women. Music therapy helps alleviate anxiety by regulating the central nervous system, while kangaroo care, through mother-infant skin-to-skin contact, promotes oxytocin secretion. The synergy between the two balances the mother's emotions, enhances her sense of safety, and fosters emotional bonding [43]. On the other hand, mindfulness-based positive psychology therapy may be more effective in improving concentration in postpartum women [44]. Kahneman's dual-system thinking model divides thought into the automatic, intuitive System 1 and the rational, deliberative System 2 [45]. Mindfulness-based cognitive training helps activate the more rational and slower System 2, reducing negative emotions triggered by System 1, thereby promoting emotional balance and enhancing concentration. Research confirms that postpartum women can demonstrably increase their attention span through effective meditation practice [46]. Compared to these psychotherapies, social support interventions—such as emotional and tangible support from partners and family members—are better suited to address the symptom of “Unhappy and cry.

The symptom “Unhappy and cry” is not only a core emotional manifestation of PPD but may also be further triggered by the arousal response of post-traumatic stress, serving a dual role as an “emotional coupling” and “activation pathway” between the two symptom clusters. Research indicates that family members can encourage postpartum women to rediscover or develop hobbies, such as painting, reading, and yoga. Through this process, positive social connections not only alleviate feelings of loneliness but also improve emotion regulation by activating the oxytocin system and prefrontal regulatory regions. This targeted intervention weakens the network influence of the bridge symptom “Unhappy and cry”, thereby enhancing creativity and enthusiasm while improving quality of life on an emotional level [47]. One study utilizing data from the Vulnerable Families and Child Welfare Study (*N* = 4150) explored the pathways between social support, stress exposure, and PPD. The results indicated that social support is an important protective factor against negative emotions in postpartum women. Therefore, social support interventions targeting the bridge symptom “Unhappy and cry” not only help alleviate negative emotions themselves but may also disrupt the emotional pathway between PPD and post-traumatic stress disorder by weakening the strength of the bridge connections. This can lead to a substantial breakdown of the comorbid network structure. Our study proposes an evidence-based hypothesis: enhancing happiness and concentration while reducing anxiety can help sever the bridge linking PPD and PP-PTSD, thereby blocking the transmission of emotional signals. Such interventions may include psychotherapeutic approaches aimed at boosting happiness and focus and alleviating anxiety, ultimately reducing the interaction between post-traumatic stress disorder and depression [35]. Future research should test this hypothesis and further explore the inter-cluster and cross-cluster functions of bridge symptoms, which may provide validated evidence for the framework of real-world clinical interventions. Additionally, longitudinal data is needed to examine the dynamic networks of postpartum women, which could offer insights into the causal relationships of how bridge symptoms affect the connection between PPD and PP-PTSD clusters.

## 6. Limitations

This study employed symptom network analysis to explore bridge symptoms between PPD and PP-PTSD, but several limitations should be noted. Given its cross-sectional design, causality between symptoms cannot be established, and the findings primarily serve as a foundation for future longitudinal and intervention studies. Additionally, the exclusion of women lost to follow-up may have led to an underestimation of symptom severity and centrality metrics. The sample for this study was drawn from a tertiary general hospital in Wuhan, which, despite its strong obstetric care capabilities and regional representativeness, may not fully capture the diverse characteristics of women in primary care settings or other regions due to differences in population demographics, healthcare-seeking behaviors, and resource allocation. Therefore, there is a clear limitation in terms of regional sample representativeness. Future research should expand the sample scope by incorporating multicenter, multilevel, and multiregional samples to enhance the robustness of the network model and improve its practical applicability.

## 7. Conclusion

This study identified several predictors for PPD included postpartum duration of 3–6 months and cesarean section. The predictors for PP-PTSD included unemployment status and household monthly income ≤3000 yuan. This study also explored the bridge symptoms and interconnections between PPD and PP-PTSD. We identified “B12 (difficulty concentrating)” and “A4 (irrational anxiety)“ as key bridge symptoms linking the two symptom clusters. Future research should adopt a longitudinal design to further investigate the dynamic symptom network in postpartum women, clarifying causal relationships, such as how bridge symptoms influence the progression of PPD and PP-PTSD. We advise that healthcare professionals pay close attention when postpartum women report severe bridge symptoms. To enhance maternal mental well-being, the psychological interventions, such as multicomponent positive psychology interventions, music therapy, kangaroo care, and social support therapy, may prove effective in alleviating these critical symptoms.

## Figures and Tables

**Table 1:** Characteristics of participants (*n* = 550).

Variables	*N* (%)
Postpartum duration	
<1 month	455 (83)
>1 year	5 (1)
3–6 months	54 (10)
6–12 months	36 (7)
Age	
<35 years	465 (85)
≥35 years	85 (15)
Education level	
Bachelor's degree	251 (46)
College	130 (24)
High school or below	72 (13)
Master's degree or above	97 (18)
Employment status	
Government employe	132 (24)
Private enterprise employe	215 (39)
Unemployed	68 (12)
Self-employed	135 (25)
Only child status	
No	337 (61)
Yes	213 (39)
Average monthly household income	
>5000	436 (79)
≤3000	22 (4)
3001–5000	92 (17)
Pregnancy intent	
Planned pregnancy	434 (79)
Unplanned pregnancy	116 (21)
Conception method	
IVF	44 (8)
Natural	506 (92)
History of miscarriage	
1 time	92 (17)
2 times	44 (8)
≥3 times	28 (5)
None	386 (70)
Pregnancy complications	
No	436 (79)
Yes	114 (21)
Fear of labor	
No	132 (24)
Yes	418 (76)
First childbirth	
No	142 (26)
Yes	408 (74)
Newborn delivery status	
Post-term	4 (1)
Premature	53 (10)
Full-term	493 (90)
Postpartum hemorrhage	
No	493 (90)
Yes	57 (10)
Newborn admitted to NICU	
No	471 (86)
Yes	79 (14)
Feeding method	
Exclusive breastfeeding	72 (13)
Mixed feeding (breast milk and formula)	336 (61)
Formula feeding	142 (26)
Baby's gender matches expectation	
No	106 (19)
Yes	444 (81)
Infant care arrangement	
Nanny	205 (37)
Mother helps	81 (15)
Mother-in-law helps	138 (25)
Self-care	126 (23)
Husband's involvement in infant care	
Poor	25 (5)
High	364 (66)
Moderate	161 (29)
Mode of delivery	
Cesarean section	341 (62)
Vaginal delivery	209 (38)
Postpartum post-traumatic stress disorder	
<13	180 (32.7%)
≥13	370 (67.3%)
Postpartum post-traumatic stress disorder	
<19	263 (47.8%)
≥19	287 (52.2%)

**Table 2:** Results of the univariate analysis on postpartum depression and postpartum post-traumatic stress disorder.

Variables	Postpartum depression (mean ± sd)	Coefficient (univariable)	Coefficient (multivariable)	Postpartum post-traumatic stress disorder (mean ± sd)	Coefficient (univariable)	Coefficient (multivariable)
Postpartum duration						
1 year (*N* = 5)	14.8 ± 3.8	0.59 (−0.52 to 1.71, *p*=0.295)	0.08 (−1.09 to 1.26, *p*=0.890)	17.6 ± 9.3	−2.81 (−5.83 to 0.21, *p*=0.068)	—
6–12 months (*N* = 36)	15.4 ± 4.7	1.26 (−0.08 to 2.60, *p*=0.065)	0.77 (−0.62 to 2.16, *p*=0.280)	21.6 ± 9.4	1.22 (−2.41 to 4.85, *p*=0.510)	—
3–6 months (*N* = 54)	20.0 ± 3.8	5.82 (2.34 to 9.29, *p*=0.001)	5.13 (1.62 to 8.65, *p*=0.004)	22.6 ± 15.5	2.24 (−7.19 to 11.66, *p*=0.642)	—
<1 month (*N* = 455)	14.2 ± 3.9	ref	—	20.4 ± 10.9	ref	—
Age						
≥35 years old (*N* = 85)	14.5 ± 3.8	0.12 (−0.80 to 1.05, *p*=0.793)	—	22.0 ± 12.9	2.09 (−0.39 to 4.56, *p*=0.098)	—
<35 years old (*N* = 465)	14.4 ± 4.0	ref	—	19.9 ± 10.2	ref	—
Education level						
College (*N* = 130)	14.1 ± 4.1	−0.42 (−1.27 to 0.42, *p*=0.326)	—	19.3 ± 10.5	−0.46 (−2.73 to 1.80, *p*=0.687)	—
Bachelor's degree (*N* = 251)	14.5 ± 4.2	ref	—	19.8 ± 10.1	ref	—
Master's degree or above (*N* = 97)	14.4 ± 3.3	−0.20 (−1.13 to 0.74, *p*=0.682)	—	21.0 ± 11.6	1.26 (−1.25 to 3.76, *p*=0.325)	—
High school or below (*N* = 72)	14.3 ± 3.9	−0.25 (−1.30 to 0.79, *p*=0.633)	—	22.2 ± 11.5	2.39 (−0.41 to 5.19, *p*=0.095)	—
Employment status						
Government employe (*N* = 132)	14.2 ± 3.9	−0.35 (−1.31 to 0.60, *p*=0.469)	—	19.1 ± 9.9	−2.62 (−5.18 to −0.05, *p*=0.046)	−3.42 (−6.02 to −0.81, *p*=0.010)
Self-employed (*N* = 135)	14.4 ± 4.0	−0.18 (−1.35 to 0.99, *p*=0.764)	—	19.4 ± 11.5	−2.35 (−5.48 to 0.78, *p*=0.140)	−3.54 (−6.72 to −0.35, *p*=0.030)
Private enterprise employe (*N* = 215)	14.3 ± 4.1	−0.23 (−1.09 to 0.64, *p*=0.607)	—	20.1 ± 10.7	−1.61 (−3.92 to 0.71, *p*=0.174)	−1.80 (−4.10 to 0.50, *p*=0.125)
Unemployed (*N* = 68)	14.6 ± 3.8	ref	—	21.8 ± 10.9	ref	—
Only child status						
No (*N* = 337)	14.3 ± 3.8	ref	—	20.1 ± 10.6	ref	—
Yes (*N* = 213)	14.5 ± 4.2	0.17 (−0.51 to 0.86, *p*=0.622)	—	20.3 ± 10.8	0.23 (−1.61 to 2.07, *p*=0.808)	—
Average monthly household income						
3001–5000 (*N* = 92)	13.9 ± 3.4	−0.40 (−2.11 to 1.31, *p*=0.645)	—	21.2 ± 10.8	1.55 (−0.84 to 3.95, *p*=0.204)	2.32 (−0.13 to 4.78, *p*=0.064)
≤3000 (*N* = 22)	14.8 ± 3.6	0.51 (−0.39 to 1.40, *p*=0.268)	—	25.5 ± 11.5	5.80 (1.24 to 10.37, *p*=0.013)	6.93 (2.30 to 11.57, *p*=0.003)
>5000 (*N* = 436)	14.3 ± 4.1	ref	—	19.7 ± 10.6	ref	—
Pregnancy intent						
Unplanned pregnancy (*N* = 116)	15.1 ± 4.2	0.86 (0.05 to 1.68, *p*=0.037)	0.71 (−0.11 to 1.52, *p*=0.049)	21.3 ± 12.3	1.38 (−0.82 to 3.57, *p*=0.218)	—
Planned pregnancy (*N* = 434)	14.2 ± 3.9	ref	—	19.9 ± 10.2	ref	—
Conception method						
Natural conception (*N* = 506)	14.5 ± 3.7	ref	—	18.9 ± 8.7	ref	—
IVF (*N* = 44)	14.4 ± 4.0	−0.16 (−1.39 to 1.07, *p*=0.802)	—	20.3 ± 10.8	1.37 (−1.93 to 4.67, *p*=0.416)	—
History of miscarriage						
No (*N* = 386)	14.4 ± 4.0	ref	—	20.0 ± 10.4	ref	—
Yes (*N* = 164)	14.4 ± 3.9	0.05 (−0.68 to 0.78, *p*=0.889)	—	20.6 ± 11.3	0.59 (−1.37 to 2.55, *p*=0.554)	—
Pregnancy complications						
No (*N* = 436)	14.4 ± 4.0	ref	—	20.1 ± 10.6	ref	—
Yes (*N* = 114)	14.2 ± 3.8	−0.18 (−1.00 to 0.64, *p*=0.670)	—	20.5 ± 11.0	0.38 (−1.83 to 2.59, *p*=0.735)	—
Fear of labor						
No (*N* = 132)	14.3 ± 4.0	−0.53 (−1.31 to 0.25, *p*=0.183)	—	19.9 ± 10.7	−1.31 (−3.40 to 0.79, *p*=0.221)	—
Yes (*N* = 418)	14.8 ± 3.8	ref	—	21.2 ± 10.6	ref	—
First Childbirth						
No (*N* = 142)	14.2 ± 3.6	ref	—	21.5 ± 11.3	ref	—
Yes (*N* = 408)	14.4 ± 4.1	0.19 (−0.57 to 0.95, *p*=0.630)	—	19.7 ± 10.4	−1.80 (−3.84 to 0.25, *p*=0.085)	—
Newborn delivery status						
Premature (*N* = 53)	14.4 ± 4.0	−0.37 (−4.30 to 3.56, *p*=0.854)	—	23.2 ± 12.5	−0.79 (−11.64 to 10.06, *p*=0.886)	—
Full-term (*N* = 493)	14.3 ± 4.1	−0.43 (−4.49 to 3.63, *p*=0.835)	—	19.8 ± 10.4	−4.17 (−14.67 to 6.34, *p*=0.436)	—
Post-term (*N* = 4)	14.8 ± 4.7	ref	—	24.0 ± 13.0	ref	—
Postpartum hemorrhage						
No (*N* = 493)	14.3 ± 4.0	ref	—	20.2 ± 10.7	ref	—
Yes (*N* = 57)	15.0 ± 4.1	0.69 (−0.40 to 1.79, *p*=0.213)	—	20.4 ± 10.9	0.28 (−2.66 to 3.22, *p*=0.852)	—
Newborn admitted to NICU						
No (*N* = 471)	14.3 ± 4.0	ref	—	20.3 ± 10.6	ref	—
Yes (*N* = 79)	14.7 ± 3.9	0.37 (−0.58 to 1.32, *p*=0.443)	—	19.7 ± 11.0	−0.59 (−3.14 to 1.96, *p*=0.650)	—
Feeding method						
Formula feeding (*N* = 142)	14.2 ± 3.8	−1.11 (−2.24 to 0.02, *p*=0.054)	−0.50 (−1.64 to 0.64, *p*=0.391)	20.5 ± 10.6	1.56 (−1.48 to 4.59, *p*=0.315)	—
Exclusive breastfeeding (*N* = 72)	14.3 ± 3.8	−1.03 (−2.04 to −0.01, *p*=0.047)	−0.51 (−1.55 to 0.53, *p*=0.334)	20.3 ± 10.9	1.38 (−1.35 to 4.11, *p*=0.321)	—
Mixed feeding (breast milk and formula) (*N* = 336)	15.3 ± 4.9	ref	—	18.9 ± 10.0	ref	—
Baby's gender matches expectation						
No (*N* = 106)	14.6 ± 4.2	ref	—	21.5 ± 10.9	ref	—
Yes (*N* = 444)	14.3 ± 3.9	−0.27 (−1.11 to 0.58, *p*=0.534)	—	19.9 ± 10.6	−1.57 (−3.83 to 0.70, *p*=0.176)	—
Infant Care Arrangement						
Nanny (*N* = 205)	14.2 ± 3.7	ref	—	20.1 ± 10.7	ref	—
Mother helps (*N* = 81)	14.4 ± 4.4	0.19 (−0.83 to 1.22, *p*=0.711)	—	21.5 ± 11.5	1.36 (−1.39 to 4.12, *p*=0.331)	—
Mother-in-law helps (*N* = 138)	14.0 ± 4.2	−0.25 (−1.11 to 0.61, *p*=0.573)	—	19.3 ± 10.5	−0.82 (−3.13 to 1.49, *p*=0.486)	—
Self-care (*N* = 126)	15.0 ± 3.9	0.75 (−0.13 to 1.64, *p*=0.094)	—	20.5 ± 10.4	0.34 (−2.04 to 2.71, *p*=0.782)	—
Husband's involvement in infant care						
Poor (*N* = 25)	16.0 ± 4.1	ref	—	20.0 ± 10.0	ref	—
Moderate (*N* = 161)	14.8 ± 4.3	−1.20 (−2.86 to 0.47, *p*=0.160)	−1.05 (−2.78 to 0.67, *p*=0.231)	20.3 ± 10.6	0.38 (−3.97 to 4.72, *p*=0.864)	—
High (*N* = 364)	14.1 ± 3.8	−1.98 (−3.59 to −0.38, *p*=0.016)	−1.61 (−3.32 to 0.09, *p*=0.064)	19.9 ± 11.0	−0.07 (−4.59 to 4.45, *p*=0.975)	—
Mode of delivery						
Cesarean section (*N* = 341)	14.8 ± 4.2	0.75 (0.06 to 1.43, *p*=0.032)	0.70 (0.02 to 1.38, *p*=0.044)	19.4 ± 9.8	−1.23 (−3.07 to 0.61, *p*=0.190)	—
Vaginal delivery (*N* = 209)	14.1 ± 3.8	ref	—	20.7 ± 11.2	ref	—
Postpartum depression	14.38 ± 3.98	—	—	—	—	—
Postpartum post-traumatic stress disorder	—	—	—	20.19 ± 10.68	—	—

*Note:p*  < 0.05 was considered statistically significant.

**Table 3:** Abbreviations and scores of postpartum depression, postpartum post-traumatic stress disorder.

No.	Item	Remark
Postpartum depression		
A1	I can laugh and see the cute side of things.	Can laugh and see cuteness
A2	I enjoy things as much as I used to.	Enjoyment remains the same
A3	I blame myself excessively when things go wrong.	Overblame myself
A4	I feel anxious and worried for no reason.	Unreasonable anxiety
A5	I feel scared and fearful without any cause.	Baseless fear
A6	I feel overwhelmed by everything.	Overwhelmed
A7	I feel unhappy and have difficulty sleeping.	Unhappy and insomnia
A8	I feel sad and miserable.	Sad and miserable
A9	I feel unhappy and cry.	Unhappy and cry
A10	I have thoughts of harming myself.	Self-harm thoughts
Postpartum post-traumatic stress disorder		
B1	Have you ever dreamed about giving birth or your child being hospitalized?	Dreamed about childbirth/hospital
B2	Have you ever had distressing memories about childbirth or your child's hospitalization?	Distress over childbirth/hospital
B3	Have you tried to avoid thinking about childbirth or your child's hospitalization?	Sudden feeling of childbirth again
B4	Have you tried to avoid thinking about childbirth or your child's hospitalization?	Avoid thinking about it
B5	Have you avoided things that remind you of childbirth or hospitalization? (e.g., TV shows)	Avoid triggers
B6	Have you ever been unable to recall some events during your child's hospitalization?	Forget events during hospitalization
B7	Have you lost interest in activities you usually enjoy? (e.g., work or family)	Lost interest
B8	Do you feel lonely or distant from others? (e.g., feeling misunderstood)	Lonely or distant
B9	Do you find it hard to feel affection or love from others?	Hard to feel love
B10	Do you have trouble falling asleep or staying asleep?	Sleep difficulties
B11	Do you feel more irritable or angry than usual?	Increased irritability
B12	Do you find it harder to concentrate than before giving birth?	Harder to concentrate
B13	Do you feel more easily startled? (e.g., sensitive to noise or easily frightened)	Easily startled
B14	Do you feel more guilty about childbirth than you think you should?	Excessive guilt over childbirth

## Data Availability

The raw data are available from the corresponding author upon request.

## References

[1] Tsai S.-S., Wang H.-H., Chou F.-H. (2020). The Effects of Aromatherapy on Postpartum Women: A Systematic Review. *Journal of Nursing Research*.

[2] Oh W., Muzik M., McGinnis E. W., Hamilton L., Menke R. A., Rosenblum K. L. (2016). Comorbid Trajectories of Postpartum Depression and PTSD Among Mothers With Childhood Trauma History: Course, Predictors, Processes and Child Adjustment. *Journal of Affective Disorders*.

[3] Wang Z., Liu J., Shuai H. (2021). Mapping Global Prevalence of Depression Among Postpartum Women. *Translational Psychiatry*.

[4] Nisar A., Yin J., Waqas A. (2020). Prevalence of Perinatal Depression and Its Determinants in Mainland China: A Systematic Review and Meta-Analysis. *Journal of Affective Disorders*.

[5] Dennis C.-L., Singla D. R., Brown H. K. (2024). Postpartum Depression: A Clinical Review of Impact and Current Treatment Solutions. *Drugs*.

[6] Oyetunji A., Chandra P. (2020). Postpartum Stress and Infant Outcome: A Review of Current Literature. *Psychiatry Research*.

[7] Bagheri P., Rostami M. (2021). Postpartum Depression and Suicide in Iran. *Women’s Health*.

[8] Yamaguchi A., Kyozuka H., Kanno A. (2021). Gestational Weight Gain and Risk Factors for Postpartum Depression Symptoms From the Japan Environment and Children’s Study: A Prospective Cohort Study. *Journal of Affective Disorders*.

[9] Adams J. A. M., Chandra P., Mehta D. (2023). The First Large GWAS Meta-Analysis for Postpartum Depression. *American Journal of Psychiatry*.

[10] Stewart D. E., Vigod S. N. (2019). Postpartum Depression: Pathophysiology, Treatment, and Emerging Therapeutics. *Annual Review of Medicine*.

[11] Harrison S. E., Ayers S., Quigley M. A., Stein A., Alderdice F. (2021). Prevalence and Factors Associated With Postpartum Posttraumatic Stress in a Population-Based Maternity Survey in England. *Journal of Affective Disorders*.

[12] Howard S., Witt C., Martin K. (2023). Co-Occurrence of Depression, Anxiety, and Perinatal Posttraumatic Stress in Postpartum Persons. *BMC Pregnancy and Childbirth*.

[13] Liu Y., Zhang L., Guo N., Jiang H. (2021). Postpartum Depression and Postpartum Post-Traumatic Stress Disorder: Prevalence and Associated Factors. *BMC Psychiatry*.

[14] Dekel S., Stuebe C., Dishy G. (2017). Childbirth Induced Posttraumatic Stress Syndrome: A Systematic Review of Prevalence and Risk Factors. *Frontiers in Psychology*.

[15] Roman M., Bostan C. M., Diaconu-Gherasim L. R., Constantin T. (2019). Personality Traits and Postnatal Depression: The Mediated Role of Postnatal Anxiety and Moderated Role of Type of Birth. *Frontiers in Psychology*.

[16] Dekel S., Ein-Dor T., Dishy G. A., Mayopoulos P. A. (2020). Beyond Postpartum Depression: Posttraumatic Stress-Depressive Response Following Childbirth. *Archives of Women’s Mental Health*.

[17] Bener A., Gerber L. M., Sheikh J. (2012). Prevalence of Psychiatric Disorders and Associated Risk Factors in Women During Their Postpartum Period: A Major Public Health Problem and Global Comparison. *International Journal of Women’s Health*.

[18] Ertan D., Hingray C., Burlacu E., Sterlé A., El-Hage W. (2021). Post-Traumatic Stress Disorder Following Childbirth. *BMC Psychiatry*.

[19] Beck-Hiestermann F. M. L., Hartung L. K., Richert N., Miethe S., Wiegand-Grefe S. (2024). Are 6 More Accurate Than 4? The Influence of Different Modes of Delivery on Postpartum Depression and PTSD. *BMC Pregnancy and Childbirth*.

[20] Pebryatie E., Paek S. C., Sherer P., Meemon N. (2022). Associations Between Spousal Relationship, Husband Involvement, and Postpartum Depression Among Postpartum Mothers in West Java, Indonesia. *Journal of Primary Care & Community Health*.

[21] Dias C. C., Figueiredo B. (2015). Breastfeeding and Depression: A Systematic Review of the Literature. *Journal of Affective Disorders*.

[22] Winstone-Weide L. K., Somers J. A., Curci S. G., Luecken L. J. (2023). A Dynamic Perspective on Depressive Symptoms During the First Year Postpartum. *Journal of Psychopathology and Clinical Science*.

[23] Koenen K. C., Ratanatharathorn A., Ng L. (2017). Posttraumatic Stress Disorder in the World Mental Health Surveys. *Psychological Medicine*.

[24] Söderquist J., Wijma B., Wijma K. (2009). The Longitudinal Course of Post-Traumatic Stress After Childbirth. *Journal of Psychosomatic Obstetrics and Gynaecology*.

[25] Horsch A., Garthus-Niegel S., Ayers S. (2024). Childbirth-Related Posttraumatic Stress Disorder: Definition, Risk Factors, Pathophysiology, Diagnosis, Prevention, and Treatment. *American Journal of Obstetrics and Gynecology*.

[26] Grisbrook M.-A., Dewey D., Cuthbert C. (2022). Associations Among Caesarean Section Birth, Post-Traumatic Stress, and Postpartum Depression Symptoms. *International Journal of Environmental Research and Public Health*.

[27] Liu X., Wang H., Zhu Z. (2022). Exploring Bridge Symptoms in HIV-Positive People With Comorbid Depressive and Anxiety Disorders. *BMC Psychiatry*.

[28] Ma H., Zhao M., Liu Y., Wei P. (2024). Network Analysis of Depression and Anxiety Symptoms and Their Associations With Life Satisfaction Among Chinese Hypertensive Older Adults: A Cross-Sectional Study. *Frontiers in Public Health*.

[29] Zhan Z.-J., Huang H.-Y., Xiao Y.-H. (2024). Anxiety and Depression in Nasopharyngeal Carcinoma Patients and Network Analysis to Identify Central Symptoms: A Cross-Sectional Study From a High-Incidence Area. *Radiotherapy and Oncology*.

[30] Cox J. L., Holden J. M., Sagovsky R. (1987). Detection of Postnatal Depression. Development of the 10-Item Edinburgh Postnatal Depression Scale. *British Journal of Psychiatry*.

[31] Zhang D., Zhang J., Gan Q. (2018). Validating the Psychometric Characteristics of the Perinatal Posttraumatic Stress Disorder Questionnaire (PPQ) in a Chinese Context. *Archives of Psychiatric Nursing*.

[32] Ayers S., Bond R., Bertullies S., Wijma K. (2016). The Aetiology of Post-Traumatic Stress Following Childbirth: A Meta-Analysis and Theoretical Framework. *Psychological Medicine*.

[33] Zhang Y., Sun H., Li W. (2021). Maternal and Paternal Depression During Pregnancy in China: Prevalence, Correlates, and Network Analysis. *Neuropsychiatric Disease and Treatment*.

[34] Ben Hayoun D. H., Sultan P., Rozeznic J. (2023). Association of Inpatient Postpartum Quality of Recovery With Postpartum Depression: A Prospective Observational Study. *Journal of Clinical Anesthesia*.

[35] Gosselin P., Chabot K., Béland M., Goulet-Gervais L., Morin A. J. S. (2016). Fear of Childbirth Among Nulliparous Women: Relations With Pain During Delivery, Post-Traumatic Stress Symptoms, and Postpartum Depressive Symptoms. *L’Encéphale*.

[36] Cho H., Koh M., Yoo H., Ahn S. (2022). Association of Postpartum Depression With Postpartum Posttraumatic Stress Disorder in Korean Mothers: A Longitudinal Survey. *Korean Journal of Women Health Nursing*.

[37] Faria-Schützer D. B., Surita F. G., Rodrigues L., Paulino D. S.de M., Turato E. R. (2020). Self-Care and Health Care in Postpartum Women With Obesity: A Qualitative Study. *Revista Brasileira de Ginecologia e Obstetrícia/RBGO Gynecology and Obstetrics*.

[38] Tang L., Zhang X., Zhu R. (2021). What Causes Postpartum Depression and How to Cope With It: A Phenomenological Study of Mothers in China. *Health Communication*.

[39] Tao Y., Hou W., Niu H. (2024). Centrality and Bridge Symptoms of Anxiety, Depression, and Sleep Disturbance Among College Students During the COVID-19 Pandemic—a Network Analysis. *Current Psychology*.

[40] Xu X., Xie T., Zhou N. (2022). Network Analysis of PGD, PTSD and Insomnia Symptoms in Chinese Shidu Parents With PGD. *European Journal of Psychotraumatology*.

[41] Cai H., Chen M.-Y., Li X.-H. (2024). A Network Model of Depressive and Anxiety Symptoms: A Statistical Evaluation. *Molecular Psychiatry*.

[42] Seshadri A., Orth S. S., Adaji A. (2021). Mindfulness-Based Cognitive Therapy, Acceptance and Commitment Therapy, and Positive Psychotherapy for Major Depression. *American Journal of Psychotherapy*.

[43] Lai H.-L., Chen C.-J., Peng T.-C. (2006). Randomized Controlled Trial of Music During Kangaroo Care on Maternal State Anxiety and Preterm Infants’ Responses. *International Journal of Nursing Studies*.

[44] Vancappel A., Hingray C., Reveillere C., El-Hage W. (2024). Disentangling the Link Between Mindfulness and Dissociation in PTSD: The Mediating Role of Attention and Emotional Acceptance. *Journal of Trauma & Dissociation*.

[45] Morewedge C. K., Kahneman D. (2010). Associative Processes in Intuitive Judgment. *Trends in Cognitive Sciences*.

[46] Prakash R. S. (2021). Mindfulness Meditation: Impact on Attentional Control and Emotion Dysregulation. *Archives of Clinical Neuropsychology: The Official Journal of the National Academy of Neuropsychologists*.

[47] Nazzal S., Ayed A., Zaben K. J., Abu Ejheisheh M., ALBashtawy M., Batran A. (2024). The Relationship Between Quality of Life and Social Support Among Pregnant Women: A Cross-Sectional Study. *SAGE Open Nursing*.

